# Individual-Based Modeling of Tuberculosis in a User-Friendly Interface: Understanding the Epidemiological Role of Population Heterogeneity in a City

**DOI:** 10.3389/fmicb.2015.01564

**Published:** 2016-01-12

**Authors:** Clara Prats, Cristina Montañola-Sales, Joan F. Gilabert-Navarro, Joaquim Valls, Josep Casanovas-Garcia, Cristina Vilaplana, Pere-Joan Cardona, Daniel López

**Affiliations:** ^1^Departament de Física, Escola Superior d’Agricultura de Barcelona, Universitat Politècnica de Catalunya–BarcelonaTechBarcelona, Spain; ^2^Departament d’Estadística i Investigació Operativa, Facultat d’Informàtica de Barcelona, Universitat Politècnica de Catalunya–BarcelonaTech, Barcelona Supercomputing Centre (BSC-CNS)Barcelona, Spain; ^3^Unitat de Tuberculosi Experimental, Fundació Institut d’Investigació en Ciències de la Salut Germans Trias i Pujol, Universitat Autònoma de BarcelonaBadalona, Spain

**Keywords:** tuberculosis, epidemiology, individual-based model, diagnosis delay, contact tracing, risk factors, HIV-tuberculosis, immigrant

## Abstract

For millennia tuberculosis (TB) has shown a successful strategy to survive, making it one of the world’s deadliest infectious diseases. This resilient behavior is based not only on remaining hidden in most of the infected population, but also by showing slow evolution in most sick people. The course of the disease within a population is highly related to its heterogeneity. Thus, classic epidemiological approaches with a top-down perspective have not succeeded in understanding its dynamics. In the past decade a few individual-based models were built, but most of them preserved a top-down view that makes it difficult to study a heterogeneous population. We propose an individual-based model developed with a bottom-up approach to studying the dynamics of pulmonary TB in a certain population, considered constant. Individuals may belong to the following classes: healthy, infected, sick, under treatment, and treated with a probability of relapse. Several variables and parameters account for their age, origin (native or immigrant), immunodeficiency, diabetes, and other risk factors (smoking and alcoholism). The time within each infection state is controlled, and sick individuals may show a cavitated disease or not that conditions infectiousness. It was implemented in NetLogo because it allows non-modelers to perform virtual experiments with a user-friendly interface. The simulation was conducted with data from Ciutat Vella, a district of Barcelona with an incidence of 67 TB cases per 100,000 inhabitants in 2013. Several virtual experiments were performed to relate the disease dynamics with the structure of the infected subpopulation (e.g., the distribution of infected times). Moreover, the short-term effect of health control policies on modifying that structure was studied. Results show that the characteristics of the population are crucial for the local epidemiology of TB. The developed user-friendly tool is ready to test control strategies of disease in any city in the short-term.

## Introduction

Tuberculosis (TB) is an infectious disease that has co-evolved with humanity with a successful strategy of remaining unperceived and acting slowly during latent infection. It is estimated that in 2013, 9 million people developed TB, and 1.5 million died from the disease (World Health Organization [WHO], 2014). To reduce TB incidence is a goal shared by many players (United Nations, 2000; Caines et al., 2006). Globally, a significant improvement at the world level has been observed since 1990. Nevertheless, there are still many challenges to face (Sulis et al., 2014). Countries with a low incidence, as is the case of most Western European states, are especially appropriate to evaluate the real viability of controlling and eliminating TB. The evolution of TB in big cities of these countries outlines many unanswered questions about the viability of the objectives. In many cases, they show incidence indexes that are much higher than the corresponding national averages.

From 1990 to 2011 different dynamics in TB indicators could be observed (de Vries et al., 2014). For instance, the incidence in London (Great Britain) increased significantly. In contrast, it decreased in cities like Barcelona (Spain), although the decreasing rate slowed down in recent years. In the Western Europe context, the current TB situation is particularly alarming. This issue is particularly the case in some cities such as London, Rotterdam, and Barcelona, among others, where heterogeneities in TB incidence between districts are evident (de Vries et al., 2014). Therefore, the present control strategies are not effective enough; there is a need for improving the procedures with new tools that serve as a test bed for assessing their effectiveness and efficiency. This improvement requires the development of mathematical models adapted to the study of TB epidemiology at the city level and, in particular, at the neighborhood level. Such models should take into account relevant factors related to social and health diversity (e.g., native/immigrant origin and HIV^+/-^, among others). At the same time, they should be designed to incorporate and test different control strategies.

Several mathematical models have been used for estimating long-term dynamics of TB epidemics (Ozcaglar et al., 2012; Zwerling et al., 2015). Most of these models have been classically built with a structured top-down strategy (Ferrer et al., 2009). In this case, the population is divided into different classes (e.g., susceptible, exposed, infected, and recovered in the case of an SEIR model) and specific fluxes are fixed between these groups. This strategy is feasible whenever the size of each class satisfies the continuum hypothesis. For instance, Ozcaglar et al. (2012) show the outcome of several simulations of an SEIR and an SEIL model with a sick compartment of 10^3^ individuals. Moreover, Castillo-Chavez and Song (2004) provide simulation results with a TB-sick population of 10^4^ individuals. Nevertheless, due to the small percentage of sick people among the infected (on average, only 10% of infected people develop an active disease), when the studied population is not big enough the resulting number of sick individuals can call the use of differential equations into question.

Furthermore, the dynamics of a TB infection inside a host depends on the particular characteristics of this host. In fact, there are several factors that have already been identified as critical for such specific dynamics. We may mention that an immunodeficiency dramatically increases the probability of developing primary active TB, and that a patient with a cavitation has a higher spreading rate than a non-cavitated patient, among others. Such heterogeneities are hard to take into account in a top-down approach, although they may have long-term consequences. Furthermore, even when considering a population with a sick fraction big enough to satisfy the continuum hypothesis, the conditions may not be homogeneous enough to make its calibration feasible. The reason lies in the diversity of possible situations of the different subpopulations due to the inherent heterogeneity of the studied system.

An alternative and complementary strategy to the top-down approach is the bottom-up perspective, i.e., the study and simulation of the parts and their interactions so that the dynamics of the global system emerge from the lowest level. This methodology is known as individual-based modelling (IbM), or agent-based modeling in a wider sense. IbM is a mathematical bottom-up approach consisting of modeling the elemental parts of the system and the relationships among them in order to obtain the behavior of the whole system (Grimm, 1999). The fundamental units of these models are the individuals, which are entities with a set of defined rules that can evolve according to them. The level of depth achieved by this kind of models allows the modeler to obtain phenomena present in the system that other models such as compartment models may overlook. Of course, the level of detail comes at the price of a bigger computational cost. However, with increasing computational capacities and the possibilities offered by high-performance computing (HPC), the drawback that this represents is notoriously compensated by the benefits of the model.

Individual-based modelling has been used in multiple fields, from simulation in social science to business, and of course in biology and epidemiology. As a consequence of this variety, IbM models have had almost as many description procedures as models developed. To deal with all the different description systems and standardize the description of these models, the overview, design concepts, and details (ODD) protocol was developed (Grimm et al., 2006, 2010). This protocol consists in formalizing the simulation model with three blocks, subdivided into seven optional subcategories: Purpose, Entities, State variables and scales, Process overview and scheduling, Design concepts, Initialization, Input data, and Submodels.

In the last decade, IbM has been introduced into the study of TB epidemiology (Montañola-Sales et al., 2015). Most of the published models were fundamentally conceived from a top-down perspective with regards to the evolution of an infected individual (i.e., an SEIR-type model extrapolated to the individual-level; Murray, 2002; Guzzetta et al., 2011; Kasaie et al., 2013, 2014). Despite their top-down approach, these works include more or less sophisticated models for the transmission routes such as taking into account the socio-demographic structure of the population and the different degrees of contact between social groups. Among these, only Guzzetta et al. (2011) dealt with a particular reality (Arkansas), while other models were fitted to global data. None of them considered relevant heterogeneity within the population, including factors like the kind of disease, possible immunodeficiency, smoking/drinking habits, and immigrant-native origin simultaneously.

The final purpose of any epidemiological model is its use by TB control units, public health agents, and politicians in order to help in decision-making. Therefore, the implementation of these models in user-friendly interfaces is a necessary step toward this end. The availability of a user interface can leverage the design of virtual experiments for testing future hypothetical situations such as the effect of immigrant flow on local TB epidemiology. Moreover, it can be used as a platform to test different control strategies and health policies. NetLogo is an agent-based programming language and integrated modeling environment that includes a user-friendly interface for scheduling simulations and performing virtual experiments (Tisue and Wilensky, 2004). Therefore, it is an adequate platform to consider when looking for a user-friendly IbM tool.

In this paper, we present an Individual-based Model of TB spreading in a community implemented in the user-friendly platform NetLogo, aiming to offer a useful tool for epidemiological study of this disease in cities. The model is described following the updated ODD protocol (Grimm et al., 2010). It has been calibrated and validated with data from Ciutat Vella (Barcelona) and used to perform a few virtual experiments to show its potential.

## ODD Description of the Individual-Based Model

### Overview

#### Purpose

The objective of this IbM is to analyze the evolution of pulmonary TB incidence in a community. It is fitted to the Ciutat Vella neighborhood, considering the population to be constant, and the possible effects of epidemiology control strategies and public health decisions are checked through virtual experiments.

#### Entities, State Variables, and Scales

The fundamental entities in the model are persons. We consider that persons can go through five infection states: healthy, infected (i.e., with a latent TB infection), sick (i.e., with an active TB), under treatment, and recovered. Persons in four out of the five states, all but healthy, are simulated as individuals. We consider that the characteristics of a healthy population remain constant (e.g., native/immigrant distribution and HIV^+^ percentage, among others). Moreover, a healthy collective is much larger than infected or sick collectives. Therefore, it is not necessary to control healthy individuals one-by-one; they are considered as a property of space (i.e., the number of healthy people in a spatial cell). A healthy person will acquire individuality once he/she enters the infection cycle. This strategy is an important optimization for drastically reducing the computing time. It was previously tested to provide results comparable to those obtained considering healthy people as individuals (Montañola-Sales et al., 2015).

**FIGURE 1:**
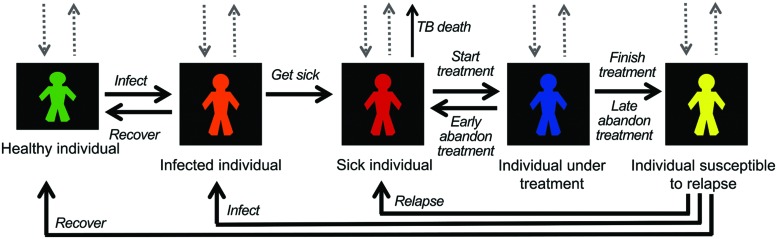
**State diagram of the model, where the five states of individuals and possible transitions are shown.** Output gray dotted arrows refer to deaths; input dotted arrows are the corresponding entrances of randomly selected individuals in order to keep population constant.

The state variables of the individuals mainly refer to their status in the TB infection cycle as well as the time spent in such phases and individual diagnostic time when getting sick. Other individual state variables and parameters are age, native/immigrant origin, risk factors (e.g., smoking), diabetes, and possible immunosuppression (mainly HIV infection). Once a person gets infected, the presence (or not) of pulmonary cavitation is also considered. A state diagram of the model is presented in **Figure 1**. The population simulated is 105,123 people, which represents all the people of Ciutat Vella (2012).

The model is partially spatially explicit, i.e., space is considered but it does not mimic the real space of Ciutat Vella. Simulation occurs in a discrete area of 501 × 501 spatial cells. Each spatial cell represents a local abstract space where two persons can meet, and the bacilli can be spread in a day. The time step is set to 1 day, and the simulation may cover up to a period of 1 or more years.

#### Process Overview and Scheduling

Our model was built in NetLogo (Tisue and Wilensky, 2004), which is well suited for modeling a broad range of agent-based systems in a user-friendly interface. The simulation starts with the set-up of the initial configuration, where the population is randomly generated according to the input distributions of parameters and randomly distributed in the 501 × 501 grid. The model assumes discrete time steps of 1 day, as mentioned. Each day, all individuals execute a series of actions, and their variables are updated immediately.

**FIGURE 2:**
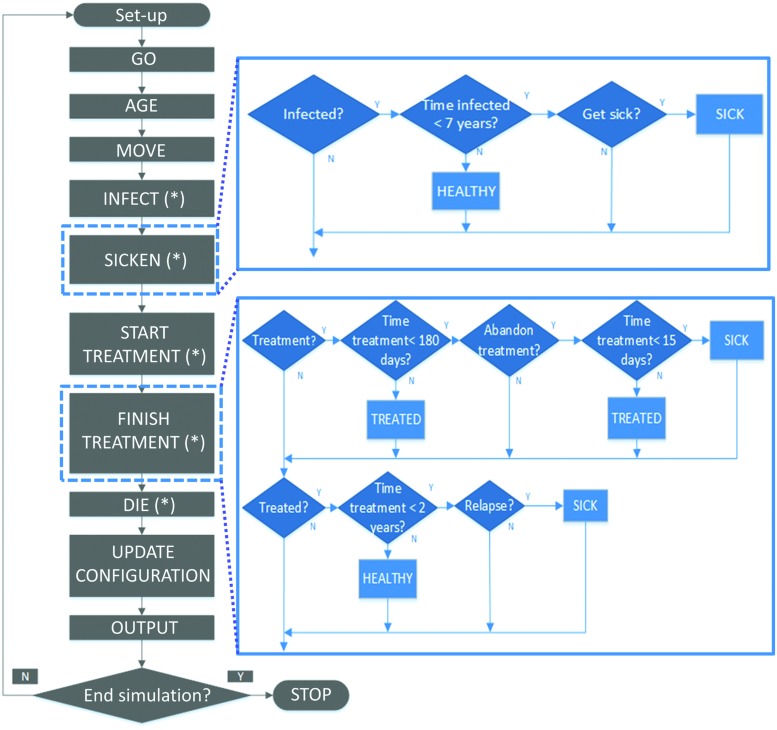
**Flow diagram of the model, with all the involved processes and subprocesses.** Boxes with an asterisk (^∗^) contain actions that can take place if specific conditions are satisfied. Details of two submodels are shown in blue (get sick and finish treatment), see the text for details of other submodels.

The individual actions may be: to age, to move, to get infected, to get sick, to be diagnosed, and start a treatment, to abandon or finish the treatment, to recover, and to die. Some of the actions take place daily for all the individuals in the system (e.g., aging and movement) and the other procedures are daily evaluated when necessary (e.g., the possibility of a sick individual to be diagnosed is daily assessed until it finally occurs). When an individual dies, a new person is introduced with particular random characteristics according to the initial distribution of individual parameters, since general population heterogeneity is assumed to remain constant during the simulation. At the end of each time, step global variables are updated. **Figure 2** shows the flow diagram of the computational model.

### Design Concepts

#### Basic Principles

The model is based on general knowledge of the natural history of TB (Cardona, 2010). There are two essential characteristics of TB that must be taken into account in any epidemiological model. On the one hand, an infected individual does not necessarily develop an active disease; on average, only 10% of infected people become sick. Moreover, a person remains infected for an extended period and may develop active TB after several years, but the probability of developing the disease decreases with time (Ferebee, 1970; Cardona and Ruiz-Manzano, 2004). Infected people are usually not diagnosed. On the other hand, only TB sick can disseminate the infection. The infection rate increases if the patient has TB with cavitation. Once a TB sick is diagnosed, the pharmaceutical treatment takes 6 months (World Health Organization [WHO], 2010). Once the treatment is finished, the possibility of getting sick again because of a TB reactivation remains at 1% for 2 years. Moreover, in some experiments a treatment for detected infected individuals is included. This treatment is longer than the one given to persons with active TB. It lasts 9 months and is administered to infected individuals to prevent the development of an active disease once they have been detected during a screening process (World Health Organization [WHO], 2015). There is also a probability of relapse to the infected state that is calculated similarly to the first treatment.

#### Emergence

Emerging phenomena are mainly related to long-term dynamics of the infection at the population level. On the one hand, only non-treated people with active TB can spread the disease. Therefore, diagnosis time is an essential parameter for the prevalence of the disease. On the other hand, infected persons may develop active TB a few years after the infection. Therefore, global consequences of particular conditions at a precise moment may be detected some years later.

#### Interaction

Local interactions between individuals are explicitly modeled and crucial for the dynamics of the system. They refer to the meeting of two persons favored by the spatial proximity between them and the possibility that one of those individuals with an active TB may infect the other person.

#### Stochasticity

Randomness is introduced at all levels of the simulation. The initial distribution of individual properties is randomly executed according to input distributions. Movement is assumed to be random. Each action is associated with a certain probability and thus executed according to a stochastic number.

#### Collectives

Two collectives may be distinguished, according to the individuals’ origin: native and immigrant. The difference between them is the diagnosis time. Due to the social patterns, it is considered that an immigrant is more liable to infect another immigrant while a native is more likely to infect another native.

#### Observation

Output data show the daily evolution of number (or prevalence) of healthy people, infected people, sick people, people under treatment, and persons already treated. These data are exported to an external data file, and an annual report is shown to the user on the interface screen.

### Details

#### Initialization

The user can change some initial conditions at the beginning of the simulation. For this particular study, most of the input parameters were taken from official reports. The initial population was fixed at 105,123 individuals. All percentages shown in **Table 1** were used for calculating the configuration of initial population: rates sick, under treatment and recovered individuals per 100,000 inhabitants; mean diagnosis delay (MDD); mean treatment abandon rate; individuals with risk factors and with HIV infection. Some other initial variables are assigned randomly: individual’s age (following the percentages shown in **Table 1**), and time spent in the infection state assigned.

**Table 1:** Official data of Ciutat Vella used in simulations (Ajuntament de Barcelona, 2012; García de Olalla et al., 2012; Bartoll et al., 2013; Orcau i Palau et al., 2013; Bartoll, 2014).

District of Ciutat Vella	Value	Units
Total population	105,123	Persons
Immigrant population	43.2%	Percentage
Population <=10 years old	7.57%	Percentage
Population 10–65 years old	77.7%	Percentage
Population > 65 years old	14.73%	Percentage
Total annual mortality	0.83%	Percentage
Detected cases of TB	64	Persons
Detected cases of TB (native)	11	Persons
Detected cases of TB (immigrant)	53	Persons
Cavitation forms^∗^	22%	Percentage
Diagnosis delay (median)	39	Days
Diagnosis delay native (median)	42	Days
Diagnosis delay immigrants (median)	33	Days
Treatment abandonment rate^∗^	2.2%	Percentage
Alive cases of VIH^+^	440	Persons
Detected cases of TB/VIH^+^	32	Persons
Risk factors^∗^	24.1%	Percentage
Diabetes cases	5.6%	Percentage

*These data correspond to Ciutat Vella demography and TB indicators in 2012, and they were used for defining the initial population and input parameters.*

*All percentages are with respect to the total population of Ciutat Vella, except (^∗^) that are with respect to the total number of TB sick people.*

#### Submodels

##### Age

All individuals increase their age by 1 day each time step.

##### Move

All persons can move randomly through the surrounding local space, once a day.

##### Get infected

If there is a number of individuals susceptible to TB (healthy and treated) different from zero in the proximity of a sick individual, meaning one of the four-neighboring spatial cells, this sick person may infect one of them with a certain probability. The total of susceptible neighboring individuals is computed and then the infection process is repeated as many times as healthy and treated people have been found. The infection probability depends on the type of TB disease that the sick person has, either cavitated or non-cavitated. A cavitation is considered to double the infection probability. The value of the infection probability is closely linked to the spatial and temporal scales, i.e., the probability of infection is inseparable from the spatiotemporal scale. A change in any of these scales entails the revision of its value. Therefore, it is not a real infection probability when a sick individual meets a healthy person, but an effective infection probability given the particular spatio-temporal constraints. In this case (501 × 501 spatial cells and 105123 individuals), the value of this probability was fixed at 49.7%. Once a person is infected, a newly infected individual is created with the properties randomly assigned according to the data from **Table 1**. Whether the new person will be set to native or immigrant will depend on the characteristics of the infective individual. Therefore, we consider that the probability of an immigrant infecting another immigrant is higher than that of infecting a native. In like manner, the likelihood of a native infecting another native is greater than that of infecting an immigrant, according to the social behavior of these communities. The infection time of the new individual is set at zero and starts increasing with each time step.

##### Get sick

Once infected, the individual may develop active TB according to a particular annual probability that decreases with infection time during the 7 years post-infection (Ferebee, 1970; Cardona and Ruiz-Manzano, 2004). It is neglected for the subsequent years (*t* > 7 years). Since simulation time does not cover periods longer than 10 years, the approximation is good enough. For immunodeficient people, a certain factor multiplies this probability. The same happens if there are other risk factors (smoking, alcoholism) or if the patient has diabetes. The chance of becoming a TB sick individual is evaluated at each time step for all infected persons. Globally, the average of 10% of infected developing an active disease is satisfied. The possibility of relapse (getting sick again) for recovered patients is also evaluated daily according to the individual relapse probability (see below). Once a person gets sick, the disease time counter starts running until the individual diagnostic time is reached.

##### Be diagnosed and start treatment

Each individual has a particular diagnostic time that is randomly assigned when getting sick. These individual times are assumed to be distributed following a normal distribution centered around the mean diagnosis time shown in **Table 1** and standard deviation 4 days. When the sick time counter reaches these values, the individual is diagnosed. Once diagnosed, medical treatment is assumed to start and TB to stop spreading. Individual time under treatment is initially fixed at zero and then updated at each time step.

##### Abandon the treatment

There is a certain probability that an individual abandons the treatment before finishing it. This possibility is evaluated daily for each patient under treatment, according to the input abandonment probability. If a person leaves treatment during the initial 15 days post-diagnosis, he/she becomes ill again. If he/she abandons the treatment after 15–180 days post-diagnosis, the model will consider him/her to be recovered but with a certain probability of relapse during the following 2 years. This probability is assumed to decrease linearly from the 100% of a 15-day abandonment to the 1% of the 180-day treatment period.

##### Recover

When a sick individual is diagnosed and treated for 180 days, he/she becomes recovered and a relapse probability of 1% is assigned (the chance of getting sick again during the following 2 years). After 2 years, the individual is considered to be healthy.

##### Die

Each individual has a certain probability of dying according to his/her age. These probabilities are fixed using demographic data from Ciutat Vella in 2012. Accordingly, the daily dying probabilities are considered to be 6.88 × 10^-5^ % for individuals under 10, 5.45 × 10^-4^% for individuals between 10 and 65, and 1.22 × 10^-2^% for individuals over 65, which is a simplification of the real mortality distribution. Furthermore, TB sick people have a distinct probability of dying from TB. This probability is evaluated daily for each sick individual, taking into account that 40% of non-treated TB sick may die in 5 years. Each time an individual dies, a new individual is introduced into the simulation world with the aim of maintaining a constant population. The individual’s characteristics are fixed according to the distribution of the initial population.

## Results

### Sensitivity Analysis and Calibration

In a first step, all the parameters that could be deduced from bibliography were fixed (**Table 1**). Then, we established minimum and maximum limits of parameters to be calibrated. We designed a series of simulations to explore the system’s behavior inside these intervals. As a result, we established the influence of these parameters on the outcome variables. Specifically, we identified which simulation output or indicator was affected the most by each parameter. For instance, the individual multiplication factor of HIV^+^/TB coinfection for developing an active disease once infected strongly determined the resulting percentage of this coinfection among TB patients. In a similar way, the multiplication factors assigned to people with diabetes or with other risk factors (smoking, alcoholism) for developing an active TB were determinant for the percentage of these collectives among TB patients. The parameter that was crucial for maintaining the reported proportion of immigrants and natives among TB patients was the probability that an infection occured within the own collective.

The most sensitive parameter was the initial number of infected people. Simulations showed that this is a critical value for the dynamics of TB among a population. Nevertheless, it is difficult, if not impossible, to determine in a real population with enough accuracy. Therefore, in this case, simulations can be used to provide an order of magnitude of such value in a particular community.

Once the sensitivity analysis was finished, the calibration process of sensitive parameters was performed using the official data of TB in 2012 (Orcau i Palau et al., 2013) as initial conditions and the official data of TB in 2013 (Orcau i Palau et al., 2015) to check the results after 1 year of simulation time. **Table 2** shows the values obtained for these parameters, together with most affected outputs of the simulation. Specifically, we display the median of 30 simulations with the parameter set obtained from the calibration and the value from the literature regarding TB in 2013. These values are relevant from the epidemiological perspective, since they provide information that is almost impossible to obtain experimentally.

**Table 2:** Values of the fitted input parameters, together with most affected outputs of the 1-year simulation compared with values in the literature (Orcau i Palau et al., 2015).

Parameter	Fitted value	Most affected output	Simulation value^1^	Literature value
Initial number of infected individuals	4500	Number of sick individuals	78.5	78
Immunodeficiency multiplication factor	18.5	Percentage of HIV/TB coinfection	6.12%	6.08%
Diabetes multiplication factor	1.2	Percentage of diabetic sick individuals	6.22 %	6.99 %
Risk-factors multiplication factor	2.0	Percentage of sick individuals with other risk factors	40.97%	40.5%
Probability that an infection affects own-collective	90%	Percentage of immigrant sick individuals	79.88%	83.33%

*^1^Median of 30 simulations.*

*These values correspond to TB indicators of Ciutat Vella in 2013.*

**FIGURE 3:**
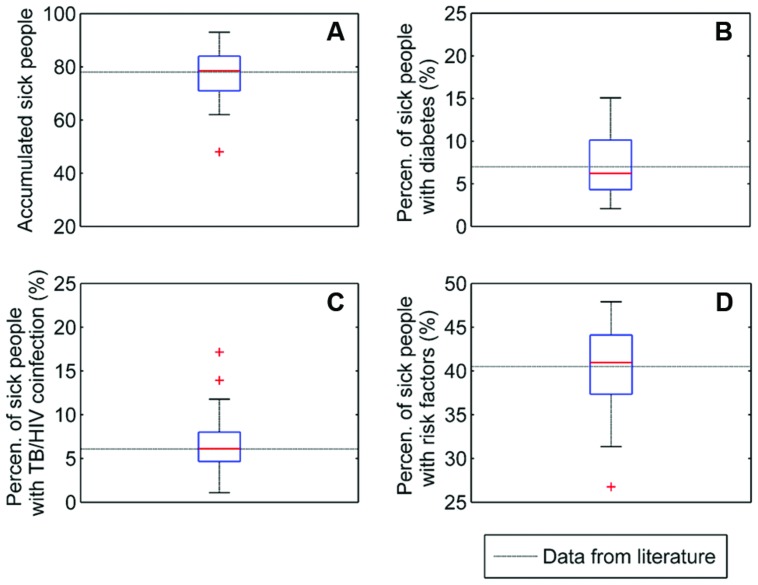
**Results of the calibration process (see **Table 2** in text).** Four relevant output of 30 runs with the calibrated dataset are shown in four boxplots: **(A)** accumulated sick people, **(B)** percentage of sick with diabetes, **(C)** percentage of sick with TB/HIV coinfection, and **(D)** percentage of sick with other risk factors. Dotted lines plot the data from the literature (Orcau i Palau et al., 2015).

**Figure 3** shows the boxplots corresponding to 30 simulation runs using the set of calibrated parameters (**Table 2**). The most affected simulation outputs are shown together with the values from the literature. These boxplots also give an idea of the inherent dispersion of simulations.

### Virtual Experiments

#### Long-Term Effect of an Increase in Diagnosis Delay Among Immigrants

In the context of the austerity policies of some governments, new regulations for accessing public health systems were introduced. For instance, on September 1, 2012, the Spanish Government approved a health care reform that explicitly excluded adult undocumented immigrants and people not paying into to social security from the public health system (BOE-A-2012-10477). Although this measure was not fully implemented by the local government in Barcelona, we used the IbM to test its hypothetical effects on TB epidemiology.

**FIGURE 4:**
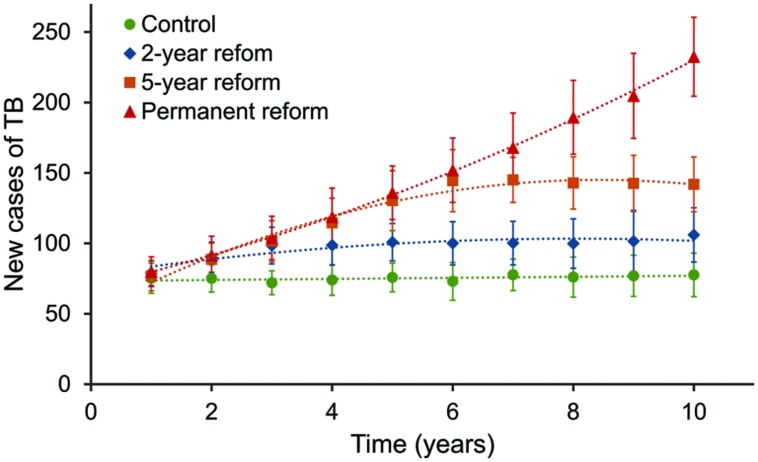
**Evolution of annual TB cases over 10 years according to virtual experiments.** Four scenarios are considered: current situation (control), in green; a 2-year health care reform, in blue; a 5-year reform, in orange; and a permanent reform, in red. The health care reform would exclude adult undocumented immigrants from the public health system and would consequently increase their diagnosis delay. We assumed the current 33-day period for non-reform cases and a 50-day period for reform cases.

We assumed an effective delay in TB diagnosis among immigrants as a direct consequence of this reform. Specifically, we examined the effect of an increase in immigrants’ mean diagnosis time up to 50 days, keeping the diagnosis time at 42 days for natives. We looked for its impact over the long-term, in a 10-year period, and considering three possible scenarios: a permanent reform (i.e., a 50-day mean diagnosis time for immigrants during the whole simulated period), 5-year transitory reform (i.e., an average diagnosis delay of 50 days for immigrants for 5 years, followed by the recovering of the current 33-day immigrant-mean diagnosis time), and a 2-year transitory reform (i.e., a MDD of 50 days for immigrants during 2 years, followed by the recovering of the current 33-day immigrant-mean diagnosis time). **Figure 4** shows the results of this experiment, comparing the annual TB cases of each situation with the control case.

A permanent reform that entailed a 52% increase in the MDD for immigrants would triple TB incidence after 10 years. Furthermore, simulation results show that the effects of a transitory reform would remain after the initial situation was restored, both for the 5 and the 2-year change. Although the restoration of initial conditions would halt the increase in TB incidence, it would take more than 10 years to get back to the TB incidence level in the control scenario. In the worst case, a permanent reform would entail not only an incidence increase, but also an increase in an accelerated way.

It would be of interest to do a rough economic evaluation of the consequences of the studied delays. According to Diel et al. (2014), the average per-case TB costs, including direct and indirect costs, in the old EU-15 states plus Cyprus, Malta and Slovenia is around €10,000. The difference in accumulated number of TB cases in 10 years between extreme simulated situations (no reform and permanent reform) is over 700. If we evaluate the worst of the cases simulated, a permanent reform that increased the MDD of immigrants up to 50 days would represent budget overruns of €7 million. This number is significant for a 10^5^ people neighborhood that would probably not compensate for the savings achieved with budget reduction. In fact, this amount could increase because of the health problems derived from a late TB diagnosis.

#### The Role of Infection Time Distribution in TB Dynamics

An IbM allows the study of a certain system at the mesoscale, i.e., between the individual (micro) and the community (macro) (Ferrer et al., 2008). A typical variable at this mesoscale is the distribution of individual properties among the population. The particular dynamics of these distributions emerge from the interaction among the parts of the system and determine the dynamics of the whole system.

We have focused on a relevant individual property: the infection time, i.e., the time elapsed from when an individual became infected. The time that people has been infected is important because the probability of developing an active disease decreases with infection time. As mentioned above, we assumed an active potential maximum infection time of 7 years. Therefore, we can talk about the distribution of infection times among population. We will use a window size of 1 year. If the global number of infected people is difficult to estimate in a real community, the time from when this infection took place is even more difficult to evaluate. An exception to this is the case in which the infection source is known.

During the calibration, we estimated the initial number of infected individuals assuming a uniform distribution, according to Occam’s razor. Nevertheless, we may wonder about potential effects of other distributions and evaluate the resulting dynamics of the system. We considered two opposite possible cases. First, the case in which in a community with a high incidence the authorities take bold measures against TB and manage to decrease the incidence remarkably. After a few years, the community would have a significant number of individuals with a long infection time and a smaller number of people with a young infection. This situation was modeled as a distribution with the shape of a positive exponential function. Second, we examined a community with a low incidence that experiences a sudden TB outbreak. This explosion could be caused for example by an immigration wave from countries with a high incidence of TB. In this situation there would presumably be a significant number of infections and the distribution would present a large number of recently infected individuals with lower levels of older infections. This situation was modeled as a distribution with the shape of a negative exponential function.

**FIGURE 5:**
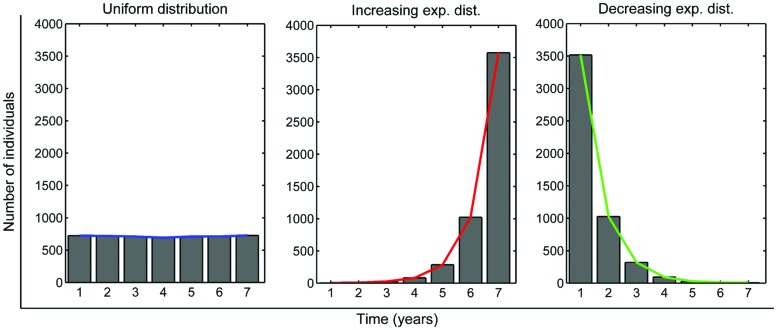
**Three infection time distributions (homogeneous, in blue; increasing exponential, in red; and decreasing exponential, in green) used as initial conditions in simulations, all corresponding to a population of 4,500 infected individuals**.

**FIGURE 6:**
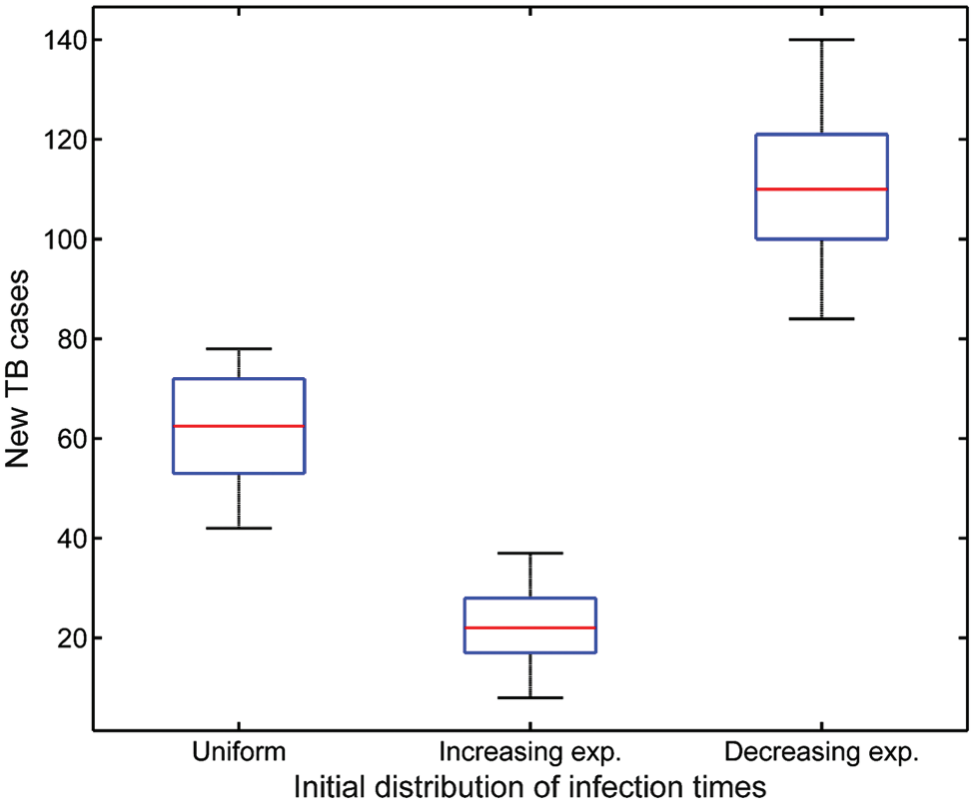
**Boxplots of virtual experiments results, showing the effect of the distribution of initial infection times (**Figure 5**) on the number of new TB cases after one simulated year**.

**Figure 5** shows the three initial infection time distributions used in simulations that would correspond to the uniform assumption and the two situations presented above. With each of these distributions, we executed 30 runs of a simulation with a duration of 1 year. At the end of the year we recorded the number of new TB cases. **Figure 6** presents a boxplot diagram of the obtained results. The most dangerous situation would be the one that lead to a decreasing exponential distribution of infection times. This outcome confirms that it is worth concentrating public health efforts in tackling new infections, in order to drastically reduce the first columns of the infection time distribution.

**FIGURE 7:**
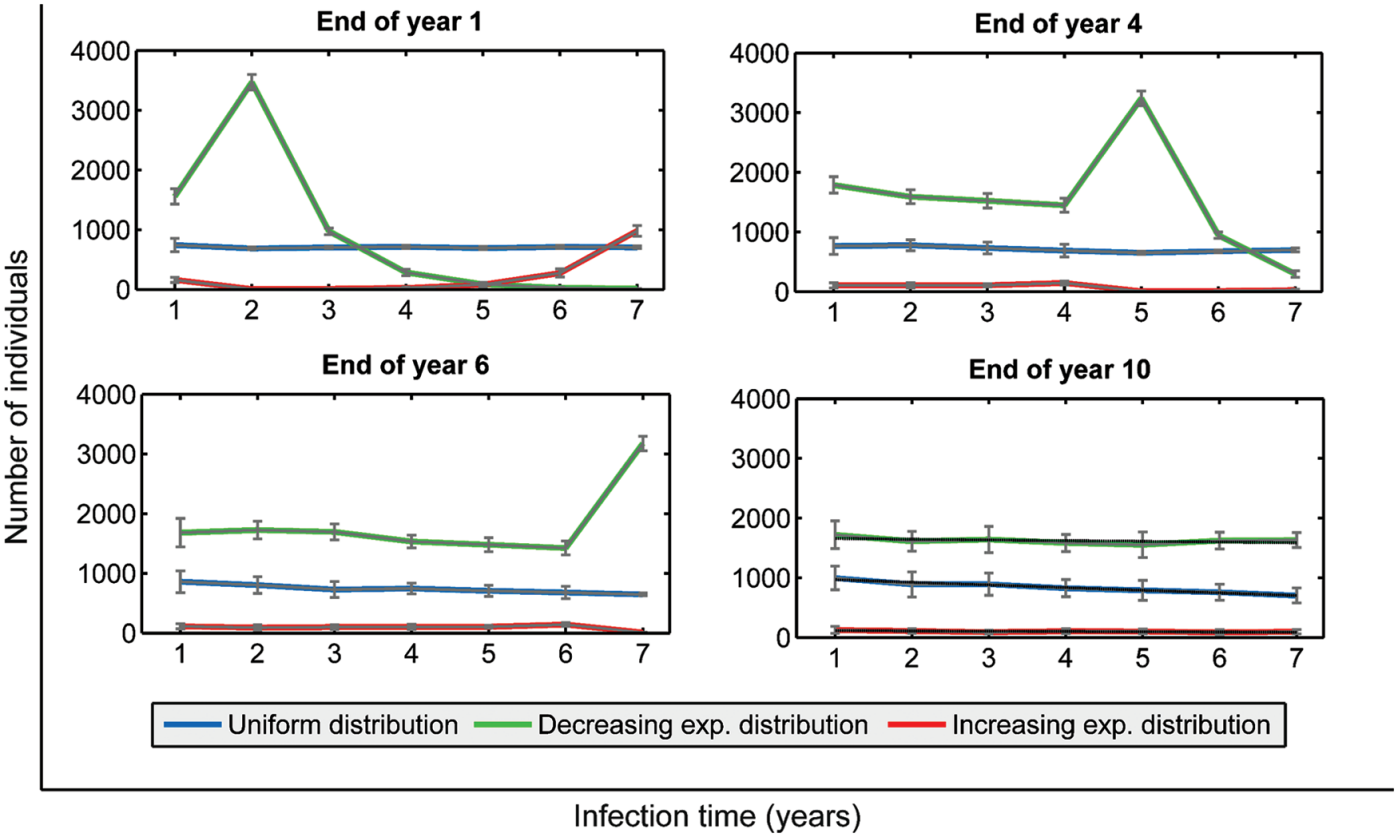
**Infection time distribution observed in virtual experiments after 1, 4, 6, and 10 years, depending on the initial infection time distribution among a 4,500 infected-subpopulation (blue: initial uniform distribution; green: initial decreasing exponential distribution; red: initial increasing exponential distribution; shown in **Figure 5**)**.

We may also observe the evolution of the infection time distributions over 10 years. As seen in **Figure 7**, all distributions evolve and lose the original shape toward a quasi-homogeneous distribution, which reveals a stationary state. In fact, all of them have a slight decreasing slope. The most significant difference between final distributions is that, although the shapes are similar, the global numbers of infected people greatly differ: an average of 5,856 infected individuals for the uniform distribution, 715 for the exponentially increasing one, and 11,367 for the exponentially decreasing one. If we compare these values with the initial amount of infected individuals (4,500), they represent a 30% increase, an 84% decrease and a 152% increase, respectively, in a constant population of 105,123 inhabitants. Since infected population is crucial for the evolution of TB in a population, as seen in the sensitivity analysis process, we may conclude that the structure of this population in terms of infection times is determinant.

#### The IbM as a Platform to Test Screenings

The goal of a screening campaign is to detect diseases before affected individuals show any symptoms. It is particularly useful in TB control due to the long time that a person may remain in the latent infection phase. There are several well-established types of screenings. Among others, we may mention mass screening, acting on the bulk of the population; selective or targeted screening, operating on a particular sector of the population that presents risk factors; and contact tracing, acting on the contacts of a diagnosed individual that would have the greatest probability of having been infected.

The objective of this virtual experiment was to determine which of different proposed screening types would be the most effective. This test also serves as a demonstration of the capabilities of the developed simulator in public health decision-making. We introduced a new sub-model to the simulator as follows.

##### Get screened

A certain amount of infected are screened, that is, identified as infected by medical authorities and undergoing preventive treatment. Therefore, these infected individuals change to a new class called treatment-screen that is equivalent to the treatment type but with a longer duration (9 months).

Then we proposed three different virtual experiments as examples to illustrate different possibilities and in order to check the model’s versatility and usefulness as a test bed, trying to cover different implementation strategies.

- *One-year contact tracing:* we assumed a hypothetical intervention only during the first year that allowed the detection of a certain percentage of the infected population. We tested hypothetical detection rates of 25, 50, 75, and 100% of the newly infected, and studied their effect at long-term.
- *Selective screening:* we selected two objective subpopulations, immigrants and individuals with HIV infection. The selective screening consisted of a one-time identification of 20% of all infected immigrants and 80% of all persons with HIV/TB co-infection. This one-time intervention was carried out in the middle of the first simulated year.
- *Permanent contact tracing:* we assumed the detection of a certain number of infected individuals each time that a TB case was diagnosed. This detection was modeled as a constant procedure during the whole simulated period. The number of detected infections per TB case was estimated from the following data corresponding to Barcelona (Millet et al., 2015): between 2009 and 2011, 541 TB cases allowed diagnosis of an extra 43 cases of TB and 1,239 TST positive cases. Therefore, we set the identification of two infected individuals per TB case diagnosed, and a 10% probability of identifying an extra TB case.

**FIGURE 8:**
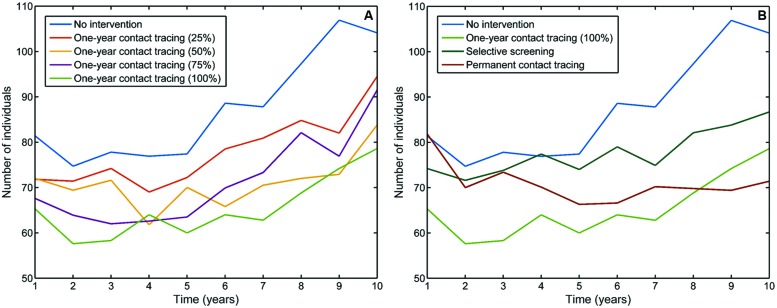
**Evolution of annual TB cases during 10 simulated years, depending on the type of screening implemented, compared with the no-intervention scenario. (A)** 1-year contact tracing with 25, 50, 75, and 100% success detecting new infections; **(B)** 1-year contact tracing with 100% success detecting new infections, selective screening (immigrants and HIV^+^), and permanent contact tracing (see details in text).

**Figure 8** shows the evolution of annual TB cases for each of the above-mentioned screenings. The 1-year variable contact tracing caused a decrease in the number of TB cases with respect to non-intervention, as expected. This decrease was particularly notable in the last years of the simulation when there was a difference of about 15 individuals for the 25% success screening rate. In the 100% success case, this difference was almost 30 cases less.

In the first years, there was also an important reduction in the number of TB cases for the one-time selective screening. However, as the simulation went on, there was an increase in TB incidence, although the results were much better than the no-intervention value.

The situation for the permanent contact tracing was utterly different. In the first years, the effect was almost negligible, but it slowly took shape by the end of the third year of simulation. From then on, the number of TB cases did not stop going down. By the end of the simulation (10 years), the number of new TB cases with the contact tracing procedure was the lowest, with a difference of more than 30 individuals from the no-intervention case.

**FIGURE 9:**
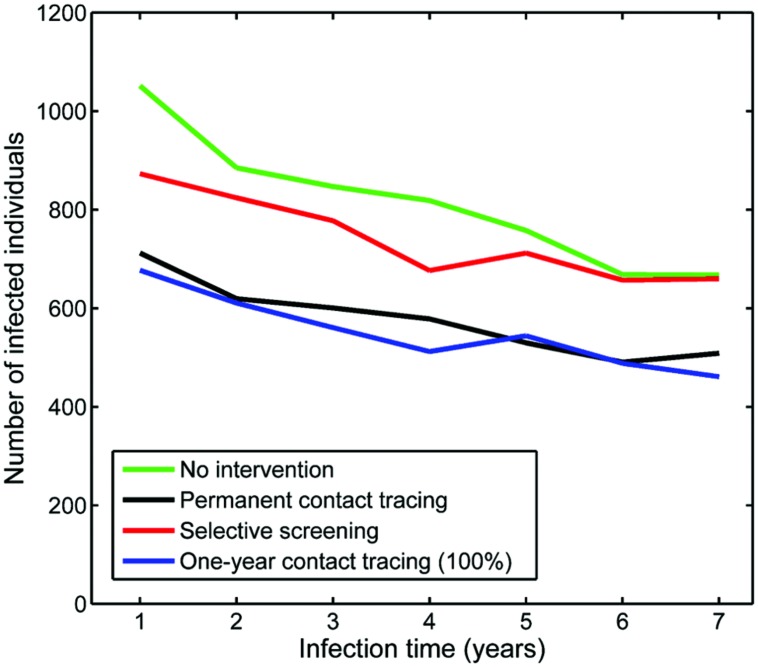
**Effect of the different tested screenings on the evolution of the infected time distribution observed in virtual experiments after 10 simulated years.** All of them started with a homogeneous distribution of 4,500 infected.

If we observe the distribution of infection times among the infected subpopulation after 10 years (**Figure 9**), we can see similar shapes but substantial differences between the global numbers of infected individuals: a final number of 5,695 infected individuals with non-intervention (26% increase with respect to initial conditions), 4,040 for permanent contact tracing (10% decrease), 4,667 for selective screening (3% increase), and 3,854 for the 100% 1-year contact tracing (14% decrease).

Although a 1-year contact tracing screening that detected 100% of the new infected people seems to provide good results that can still be perceived over the long-term, this is an entirely unrealistic scenario. In fact, selective screenings and permanent (partial) contact tracing are strategies that are currently being implemented in some cities like Barcelona. With the conditions imposed to simulations, the permanent contact tracing would provide better longstanding results. The apparently irrelevant effect of selective screening in these simulations can be explained by two factors. Communities in which it is applied are subpopulations; therefore, global consequences are not outstanding. In this case we assumed a single screening while contact tracing was carried out each time a new TB case was diagnosed. At this point we should mention that all the screenings simulated were not designed from a real application point-of-view, but with an experimental perspective to test the potential of this IbM. In order for it to be useful in decision-making, the precise interventions to be incorporated should be designed in collaboration with health control specialists and adapted to the specific case to be tackled.

## Discussion

We have developed an IbM for TB spreading at the city-level. This simulator was implemented in the user-friendly platform NetLogo with the aim of facilitating its use by non-modelers. It was calibrated and validated with available data from Ciutat Vella, a neighborhood in Barcelona with an incidence of 67 TB cases per 100,000 inhabitants in 2013. Three virtual experiments were used for testing its potential, mainly focused on the population heterogeneity.

The first virtual experiment was aimed at an especially important subpopulation of Ciutat Vella: immigrants. We showed that an increase in the MDD of this collective would revert to the whole population TB dynamics, the effects persisting over the long-term. The second virtual experiment aimed to explore the importance of the structure of the infected subpopulation with regards to the infection time. Keeping the initial number of infected individuals, the shape of this distribution crucially directs the dynamics of TB. The third virtual experiment was designed to test the platform as a tool for helping decision-making. It quickly allowed the incorporation of strategies directed either to the global community or specific collectives, as well as the analysis of the consequences for the structure of the infected subpopulation. In fact, the second and third experiments demonstrated the importance of the hidden infected collective in the dynamics of the TB disease. Thus, it seems essential to keep directing efforts to the control of this population cohort.

Beyond the results of the specific cases reported in this paper, we want to emphasize the methodology used in this approach. Mathematical models in epidemiology are used to improve understanding and for predictive purposes. Historically, these models have been expressed mainly with differential equations. Continuous models are especially useful for studying the spreading of diseases that follow the ecological r-strategy, i.e., with a rapid increase in their incidence (Fierer et al., 2007). This strategy usually guarantees that the fraction of population simultaneously affected in a small period is large enough to be statistically significant and to support the continuum hypothesis. Moreover, this also guarantees that short-term predictions are enough, which is important because environmental and social conditions that affect the dynamics of a disease spreading may be difficult to control over long-term periods. In contrast, we can talk about diseases that follow the ecological K-strategy (Fierer et al., 2007), i.e., with slow dynamics and small incidence but greater persistency in a community, usually remaining hidden for years. In this case, the use of continuous models may be controversial, mostly because of the limited size of the simultaneously affected population.

Tuberculosis may be considered a K-strategy disease. As such, the number of people with a simultaneous active disease in a certain community is relatively small. The alternative of increasing the modeled population is not feasible since the inherent heterogeneity of situations also complicates the parameterization and interpretation of results. The modeling strategy that we have proposed in this paper is the use of an Individual-based approach to studying TB spreading in communities such as neighborhoods or cities. On this scale, population characteristics and the socio-economic environment can be delimited and controlled. This approach allows proper definition of individuals’ behavior with simple rules, as well as the possibility of reproducing the diversity of possible situations.

The interest of the output of such models is the study of variations in global dynamics emerging from actions over this community. In fact, the reality is complex and dynamic, and a wide variety of unexpected socio-economic changes that may influence the epidemics of a disease like TB can occur. Thus, IbM simulations should not aim to predict future evolution from a realistic perspective, but rather to provide a virtual platform to test different situations. Questions like the effect of migration fluxes with different infection profiles, the impact of variations on certain acting protocols, and the effect of changes in social habits due to specific socio-economic situations, among others, can be addressed one-by-one (i.e., each independently from the others) or in combination. In general, the IbM simulations designed to answer these issues require just a few changes in the model and the computer code. The answers to these questions should help the healthcare community and improve policy decision-making.

## Author Contributions

CP co-leaded this research project, designed the model and the virtual experiments, supervised model’s implementation, carried out the virtual experiments, analyzed and discussed the results and leaded the writing of the manuscript. CM-S designed and supervised model’s implementation, designed the virtual experiments, analyzed and discussed the results and co-leaded the writing of the manuscript. JG implemented the model, carried out the virtual experiments, analyzed the results and participated in the writing of the manuscript. JV and JC-G designed and supervised the model’s implementation, analyzed and discussed the obtained results and critically revised the manuscript. CV and P-JC designed the model and the virtual experiments, analyzed and discussed the obtained results and critically revised the manuscript. DL conceived and leaded this research project, designed the model and the virtual experiments, analyzed and discussed the obtained results and participated in the writing of the manuscript.

## Conflict of Interest Statement

The authors declare that the research was conducted in the absence of any commercial or financial relationships that could be construed as a potential conflict of interest.
